# Direct exposure to mild heat promotes proliferation and neuronal differentiation of neural stem/progenitor cells *in vitro*

**DOI:** 10.1371/journal.pone.0190356

**Published:** 2017-12-29

**Authors:** Md Emon Hossain, Kentaro Matsuzaki, Masanori Katakura, Naotoshi Sugimoto, Abdullah Al Mamun, Rafiad Islam, Michio Hashimoto, Osamu Shido

**Affiliations:** 1 Department of Environmental Physiology, Faculty of Medicine, Shimane University, Enya-cho, Izumo, Japan; 2 Department of Nutritional Physiology, Faculty of Pharmaceutical Sciences, Josai University, Sakado, Saitama, Japan; 3 Department of Physiology, Graduate School of Medical Science, Kanazawa University, Kanazawa, Japan; Lewis Katz School of Medicine at Temple University, UNITED STATES

## Abstract

Heat acclimation in rats is associated with enhanced neurogenesis in thermoregulatory centers of the hypothalamus. To elucidate the mechanisms for heat acclimation, we investigated the effects of direct mild heat exposure on the proliferation and differentiation of neural stem/progenitor cells (NSCs/NPCs). The NSCs/NPCs isolated from forebrain cortices of 14.5-day-old rat fetuses were propagated as neurospheres at either 37.0°C (control) or 38.5°C (mild heat exposure) for four days, and the effects on proliferation were investigated by MTS cell viability assay, measurement of neurosphere diameter, and counting the total number of cells. The mRNA expressions of heat shock proteins (HSPs) and brain-derived neurotrophic factor (BDNF), cAMP response element-binding (CREB) protein and Akt phosphorylation levels, and intracellular reactive oxygen species (ROS) levels were analyzed using real time PCR, Western blotting and CM-H_2_DCFDA assay respectively. Heat exposure under proliferation condition increased NSC/NPC viability, neurosphere diameter, and cell count. BDNF mRNA expression, CREB phosphorylation, and ROS level were also increased by heat exposure. Heat exposure increased HSP27 mRNA expression concomitant with enhanced p-Akt level. Moreover, treatment with LY294002 (a PI3K inhibitor) abolished the effects of heat exposure on NSC/NPC proliferation. Furthermore, heat exposure under differentiation conditions increased the proportion of cells positive for Tuj1 (a neuronal marker). These findings suggest that mild heat exposure increases NSC/NPC proliferation, possibly through activation of the Akt pathway, and also enhances neuronal differentiation. Direct effects of temperature on NSCs/NPCs may be one of the mechanisms involved in hypothalamic neurogenesis in heat-acclimated rats. Such heat-induced neurogenesis could also be an effective therapeutic strategy for neurodegenerative diseases.

## Introduction

Heat acclimation (HA) is an adaptive physiological process that increases heat tolerance [1, 2]. Heat-acclimated animals exhibit various physiological changes, especially in the thermoregulatory and cardiovascular systems, such as enhanced sweating and cutaneous vasodilation, increased plasma volume, and reduced heart rate [1, 2]. Depending on the duration of heat stimuli, the process of heat acclimation is classified into two types, i.e., (1) short-term HA (STHA), and (2) long-term HA (LTHA) [3]. In rodents, STHA requires 5–6 days of heat exposure while LTHA requires 4–5 weeks [4, 5]. Physiological changes due to STHA rapidly disappear after heat exposure is withdrawn, while the changes associated with LTHA are stable and sustained for a prolonged period [4, 5].

Thermal homeostasis in humans and rodents is regulated mainly by hypothalamic areas of the brain. The preoptic area of the hypothalamus is widely regarded as the principal thermoregulatory region [6–11]. However, other hypothalamic areas are also believed to be involved in modulating thermoregulatory afferent and efferent signals, which contribute to monitor core and skin temperatures and control the thermoeffectors in the peripheral regions [6–11]. Since LTHA is long lasting, the process might cause functional and morphological changes in the hypothalamic thermoregulatory centers to achieve such persisting effect. Hence, several studies have been performed focusing on changes in gene expression profile [12, 13], morphological characteristics of neurons and synaptic structures [14] in the anterior hypothalamus. Notably, the ratio of hypothalamic thermosensitive to thermo-insensitive neurons is changed after heat exposure, suggesting a considerable plasticity exist in thermoregulatory centers [15]. Such plasticity could be involved in establishing LTAHAHhhHA, although the exact cause that changes the ratio of neuronal types is unknown.

It is well established that neurogenesis is maintained in the adult mammalian brain in both the subventricular zone of the lateral ventricles and the hippocampal subgranular zone [16, 17]. Recent reports suggest that neurogenesis also occurs in the hypothalamus in response to various external stimuli [18–20]. Neural stem/progenitor cells (NSCs/NPCs) were demonstrated to proliferate in the ependymal layer of the third ventricle, migrate to hypothalamic parenchyma, differentiate into mature neurons, and functionally integrate into neural networks [18, 20]. Such findings prompted us to investigate the role of neurogenesis in the process of HA. In previous studies, we found that constant moderate heat exposure for 5 days increased NSC/NPC proliferation in rat hypothalamus [21–24]. Moreover, if heat exposure was continued for several weeks, the newly generated NSCs/NPCs migrated into the hypothalamic parenchyma, differentiated into neurons, and were incorporated into neural circuits [21]. In LTHA, incorporation of newly generated neurons could rearrange the circuitry in thermoregulatory center. In addition, we recently reported that inhibition of NSC/NPC proliferation by a mitotic blocker, cytosine arabinoside, decreased heat tolerance in rats [23]. Thus, NSC/NPC proliferation and integration into hypothalamic neural circuitry may be important for acquired HA. However, the exact mechanisms of heat exposure-induced NSC/NPC proliferation in rat hypothalamus are unclear.

Heat exposure elevates core body temperature [21]. High body temperature physically facilitates biological reactions due to the temperature coefficient (*Q*_10_) effect and may thereby accelerate cell proliferation. However, the effects of direct heat exposure on neural stem/progenitor cell (NSC/NPC) proliferation have not been investigated. Numerous studies have reported growth stimulation of various cell types by direct heat exposure. For example, heat exposure has been shown to induce cyclin D1 in NIH3T3 cells, indicating a stimulatory effect of temperature on cell proliferation [25]. The T-cell proliferative response to interleukin-1 is also greatly increased at 39.0°C compared with that at 37.0°C [26]. Heat exposure induces the proliferation and differentiation of bone marrow-derived stromal cells, suggesting that the direct effects of temperature on bone-forming cells may be involved in heat-induced bone formation [27]. Based on these findings, we speculated that elevated core body temperature may directly accelerate the proliferation rate of NSCs/NPCs in rat hypothalamus. To test this hypothesis, we cultured NSCs/NPCs under normal and elevated temperatures, and examined whether direct heat exposure exerts stimulatory effects on NSC/NPC proliferation. Heat exposure initiates various cellular responses in cells, including induction or activation of heat shock proteins (HSPs) [28]. Some of these HSPs, such as HSP90, are constitutively expressed, while others, particularly HSP27 and HSP70, are inducible [29, 30]. In addition to their functions as molecular chaperones, both HSP27 and HSP70 have been shown to confer cytoprotection against apoptosis [31, 32] and necrosis [33–35] by a variety of stressors. Further, HSP27 can interact with the Ser/Thr kinase Akt which is suggested to be important for sustained Akt activity [36–38] and Akt has been reported to mediate promotion of cell proliferation and cell survival [39]. Thus, in order to elucidate the mechanism of heat-induced NSC/NPC proliferation, we investigated HSPs induction and Akt activation in heat-exposed NSCs/NPCs. We also examined whether neuronal differentiation of cultured NSCs/NPCs is enhanced by direct heat exposure.

## Materials and methods

### Ethics statement

All animal experiments were performed in accordance with the Guidelines for Animal Experimentation of Shimane University Faculty of Medicine, which were compiled from the Guidelines for Animal Experimentation of the Japanese Association for Laboratory Animal Science. The protocol for this study was approved by the Committee on the Ethics of Animal Experiments of Shimane University (Permit Number: IZ 27–18). All surgeries were performed under anesthesia, and all efforts were made to minimize suffering and the number of animals used. The rats were anesthetized by intraperitoneal injection of a mixture of medetomidine hydrochloride (0.15 mg/kg), midazolam (2.0 mg/kg), and butorphanol tartrate (2.5 mg/kg). Carbon dioxide (CO_2_) inhalation was used for euthanasia of pregnant rats. Isolation of NSCs/NPCs from forebrain cortices required decapitation of fetuses with surgical scissors.

### Isolation and culture of fetal NSCs/NPCs

NSCs/NPCs were isolated from rat fetuses on embryonic day 14.5 (E14.5) and cultured by the neurosphere method as previously described [40, 41]. Briefly, rat forebrain cortices were dissected on E14.5 and mechanically dissociated into single cells by repeated pipetting (trituration) in a serum-free medium (N2 medium) containing DMEM/F12 (1:1), 0.6% (w/v) glucose, 0.1125% (w/v) sodium bicarbonate, N2 supplement, 2 mM L-glutamine, 5 mM HEPES, and 25 μg/mL insulin. The dissociated cells were cultured in 60-mm dishes at a density of 5 × 10^5^ cells per dish in N2 medium supplemented with 20 ng/mL basic fibroblast growth factor (bFGF) and 2 μg /mL heparin (proliferation media, PM) in a humidified 5% CO_2_/ 95% air incubator at 37.0°C. Within 3–5 days, the cells propagated as free-floating neurospheres that were collected by centrifugation, mechanically dissociated into single cells, and then passaged twice. Most cells dissociated from neurospheres were positive for the NSC/NPC markers, nestin and prominin-1 (CD133), whereas a small number were positive for neuron-specific class III beta-tubulin-(Tuj-1-) and glial fibrillary acidic protein (data not shown).

For preparation of adherent monolayer NSC/NPC cultures, neurospheres were dissociated into single cells, which were then seeded in PM supplemented with 1% FBS to facilitate attachment. Most of the cells in monolayer culture were positive for sex determining region Y-box 2 (SOX2) (data not shown).

### Cell proliferation assays

**Fig 1:**
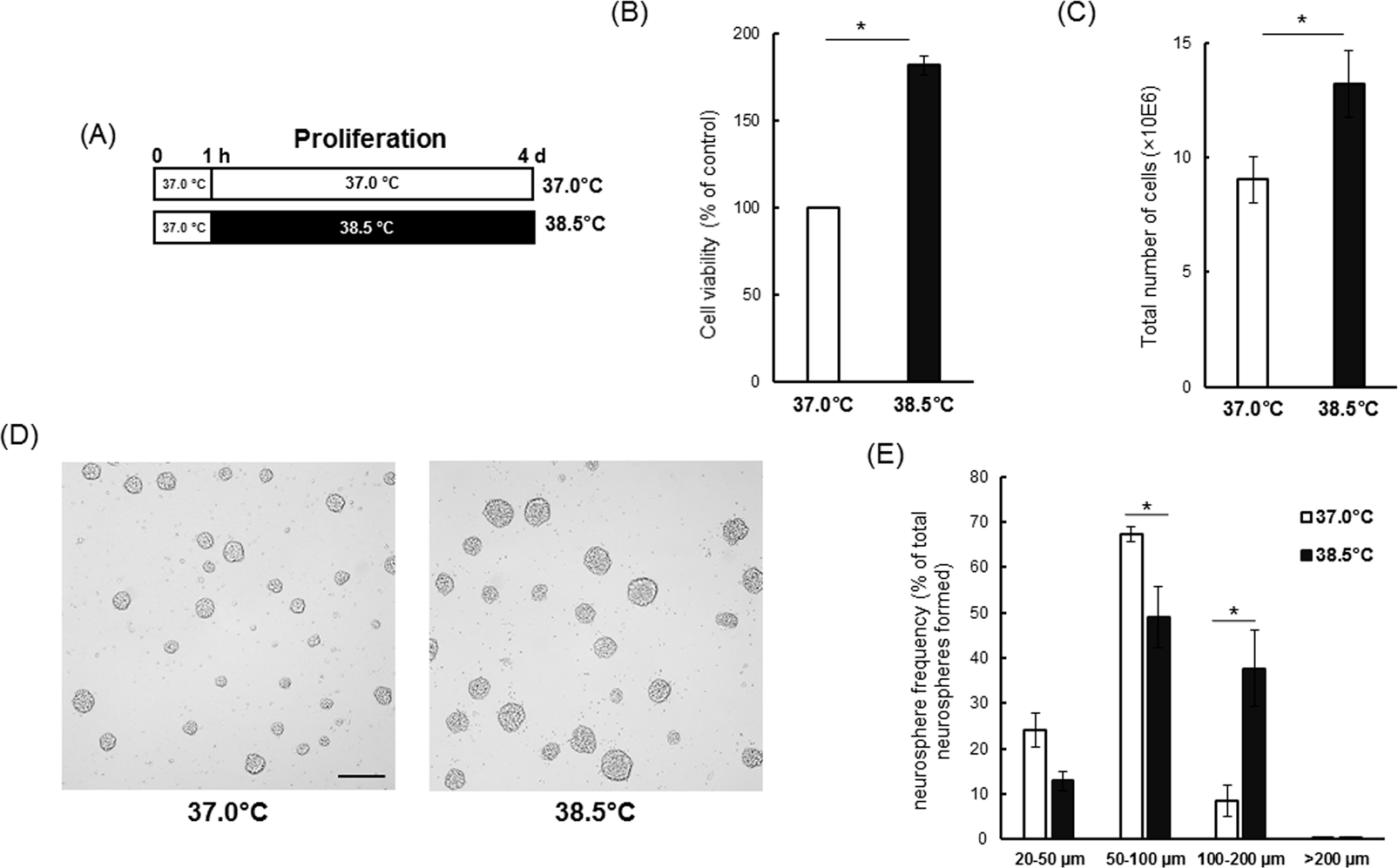
Mild heat exposure accelerates neural stem/progenitor cell proliferation rate under proliferation culture conditions. (A) NSCs/NPCs were cultured in proliferation media (PM) at either 37.0°C or 38.5°C for 4 days. (B) On day 4 of culture in PM, viability was measured by MTS cell viability assay. Data are expressed as percentages of the control temperature group (37.0°C). Results are mean ± SEM of five independent experiments. *P < 0.05 vs. control temperature. (C) On day 4, neurospheres generated at control temperature and under mild heat exposure were dissociated separately by trituration and the total number of cells counted by trypan blue exclusion. Values are mean ± SEM of five independent experiments. *P < 0.05 vs. control temperature. (D) Representative images of neurospheres formed at 37.0°C or 38.5°C on day 4 of proliferation. Bar indicates 250 μm. (E) Quantitative analysis of neurosphere size distribution arbitrarily divided into four classes according to diameter. Data are expressed as percentages of the total number of neurospheres. Results are mean ± SEM of four independent experiments. *P < 0.05 vs. control temperature.

After dissociating the neurospheres, single cells were cultured in PM at a density of 0.5 × 10^5^ cells/mL in 96-well plates or 60-mm dishes under different conditions of heat exposure as indicated in Fig 1A and S1 Fig. The cells were grown as neurospheres for four days. On day 4, cell proliferation was assessed by determining cell viability, neurosphere size, and cell count under each condition.

#### Cell viability

Cell viability was assessed using the CellTiter 96 AQueous One Solution Assay (MTS assay; Promega, Madison WI, USA). On day 4 of culture, all 96-well plates (treated as indicated) were incubated at 37.0°C for 1 h and then 10 μL of the AQueous One Solution reagent was added directly to each well. After 3 h of incubation at 37.0°C, the absorbance at 490 nm was measured on a microplate reader. The absorbance at 490 nm is directly proportional to the number of living cells in each well.

#### Cell counting

The neurospheres generated in 60-mm dish under each condition were collected by centrifugation and mechanically dissociated into single cells by repeated pipetting. The single cells per dish were counted by the trypan blue exclusion method.

#### Neurosphere size analysis

On day 4 of culture, neurosphere diameter was measured automatically in 96-well plates using the IN Cell Analyzer 1000 System (GE Healthcare Biosciences). Only neurospheres larger than 20 μm in diameter were analyzed.

### 5-Bromo-2'-deoxyuridine (BrdU) pulse labeling

Short-term (pulse) BrdU labeling was performed, as described [42], with slight modifications. Briefly, NSCs/NPCs were cultured in PM supplemented with 1% FBS at 37.0°C or 38.5°C. On day 3, 10 μM BrdU (BD Biosciences, San Jose CA, USA) was added for 4 h before fixation of cultures with 4% paraformaldehyde. Cells were washed with phosphate-buffered saline (PBS) containing 50 mM glycine and then incubated in 2N HCl for 10 min at 37.0°C. The HCl was replaced with 0.1 M borate buffer and incubated for 10 min at room temperature before immunostaining with rat anti-BrdU antibody (BIO-RAD, Hercules, CA, USA) and counterstaining with 4′,6-diamidino-2-phenylindole dihydrochloride (DAPI; 1:3000; Dojindo Molecular Technologies). The numbers of BrdU-positive cells and total cells were counted in seven random fields per well.

### LY294002 treatment

For PI3K inhibition experiments, 10 μL of LY294002 (abcam, Cambridge UK), supplied in dimethyl sulfoxide (DMSO), was directly added to the wells on day 1 at various final concentrations ranging from 0 to 50 μM, and cell viability was measured by MTS assay on day 4. Control cells were treated with vehicle (DMSO) at 37.0°C. To avoid any nonspecific toxic effects of DMSO on cell growth, DMSO concentrations were maintained at <0.5% in all experiments.

### Differentiation of NSCs/NPCs

To differentiate NSCs/NPCs, we collected proliferating neurospheres growing at 37.0°C or 38.5°C separately and mechanically dissociated them into single cell populations. The single cells were washed once and resuspended in N2 medium. The cells were then seeded onto 24-well plates coated with poly-L-ornithine (15 μg/mL; Sigma-Aldrich, St. Louis MO, USA) at a density of 2 × 10^5^ cells/mL. After 1 h incubation at 37.0°C, the plates were incubated under 5% CO2 at 37.0°C or 38.5°C for 4 days.

### Immunofluorescence staining

On day 4 of differentiation, cells were fixed with 4% paraformaldehyde for 30 min at room temperature, washed with 0.1 M Tris-buffered solution (TBS; pH 7.5), blocked with 3% normal goat serum (Dako) in TBS containing 0.3% Triton X-100 at room temperature for 60 min, and incubated overnight with a mouse primary antibody against the neuronal marker Tuj1 (1:1000; R&D Systems, Minneapolis MN, USA) at 4°C. Cells were then washed with TBS and incubated with Alexa Fluor 488-conjugated secondary antibody (1:1000; Invitrogen, Carlsbad, CA, USA) at room temperature for 60 min. To visualize nuclei, the cells were counterstained with 4',6-diamidino-2-phenylindole dihydrochloride (DAPI; 1:3000; Dojindo Molecular Technologies, Inc.). Finally, stained cells were mounted with 80% glycerol and visualized under a fluorescent laser microscope (FV1000D; Olympus). Images were processed using ImageJ software (NIH, USA). The numbers of Tuj1-positive and total cells were counted in seven random fields per well.

### Western blot analysis

All primary antibodies (rabbit anti-phospho Akt^Ser473^, rabbit anti-phospho-CREB, mouse anti-CREB, and rabbit anti-β-actin) and secondary antibodies (goat anti-rabbit IgG and horse anti-mouse IgG) for western blot analysis were purchased from Cell Signaling Technology (Danvers, MA, USA). On days 1 and 4 of proliferation, cells were collected, washed with PBS (pH 7.6), and lysed in RIPA buffer (Cell Signaling Technology) containing 20 mM Tris-HCl (pH 7.5), 150 mM NaCl, 1 mM Na_2_EDTA, 1 mM EGTA, 1% NP-40, 1% sodium deoxycholate, 2.5 mM sodium pyrophosphate, 1 mM β-glycerophosphate, 1 mM Na_3_VO_4_, 1 μg/ml leupeptin, and protease inhibitor cocktail (Roche, Basal, Switzerland). After incubation on ice for 30 min, the supernatant was collected by centrifugation at 13,000 rpm for 15 min at 4°C and the protein concentration of the supernatant was determined using the Pierce BCA protein assay kit (Thermo Scientific). Protein samples (10 μg) from each treatment group were separated on 10% Tris-HCl denaturing gels by SDS-PAGE and transferred to polyvinylidene difluoride membranes (Bio-Rad). Membranes were blocked with 5% skim milk (Wako) and incubated with primary antibodies (1:1000) overnight at 4°C followed by five washes in Tris-buffered saline (TBS) containing 0.1% Tween 20. The membranes were then incubated with a horseradish peroxidase (HRP)-conjugated anti-rabbit or anti-mouse secondary antibody (1:2000) for 1 h at room temperature and then washed five times with TBS. The HRP was detected by Amersham ECL Prime western blotting detection reagent (GE Healthcare) and visualized using an Image Quant LAS-4000 biomolecular imager (GE Healthcare).

### Real-time PCR

The NSCs/NPCs were allowed to grow in PM for the indicated times and collected. Total RNA was isolated using Isogen reagent (Wako), reverse transcribed into cDNA using the QuantiTect Reverse Transcription Kit (Qiagen), and amplified using the ABI prism 7000 sequence detection system (Applied Biosystems). Real-time polymerase chain reaction was carried out with the QuantiTect SYBR Green PCR Kit (Qiagen). The primer sequences are listed in S1 Table. The PCR conditions were as follows: initial activation at 95°C for 15 min, then 40 amplification cycles of denaturation at 94°C for 15 s, annealing at 58°C for 30 s, and extension at 72°C for 30 s. Relative quantification was performed using the 2^−ΔΔCt^ method with 18S rRNA as the endogenous control. Relative gene expression is presented as a ratio of target gene expression under mild heat exposure to expression at the control temperature.

### Measurements of intracellular reactive oxygen species (ROS)

Intracellular ROS level was measured using the oxidant-sensitive fluorescent probe 5-(and-6)-chloromethyl-2',7'-dichlorodihydrofluorescein diacetate acetyl ester (CM-H_2_DCFDA; Molecular Probes, Eugene OR, USA). NSCs/NPCs were cultured at 37.0°C or 38.5°C. On day 1 and 4 of proliferation culture, the medium was removed and cells were suspended at 1 × 10^6^ cells/ml in culture medium containing CM-H_2_DCFDA (10 μM) for 30 min at 37.0°C. The cells were then washed with Hank’s balanced salt solution to remove excess extracellular CM-H_2_DCFDA and lysed in 0.1% Triton X-100. The supernatant was collected by centrifugation at 13,000 rpm for 15 min at 4°C. Supernatant samples (200 μl) were placed in 96-well black plates and fluorescence signals were measured using a fluorescence microplate reader at an Ex/Em: 485/535 nm (DTX 880 multimode detector, Beckman Coulter, Inc.). Protein concentration of the supernatant was determined using the Pierce BCA protein assay kit (Thermo Scientific).

### Statistical analysis

All data are expressed as mean ± SEM. Means were compared by Student’s paired t-tests. For multiple comparisons, one-way ANOVA was performed followed by Tukey post-hoc tests. Alpha values were 0.05 except when adjusted by the post-hoc tests.

## Results

### Mild heat exposure increased proliferation of NSCs/NPCs

**Fig 2:**
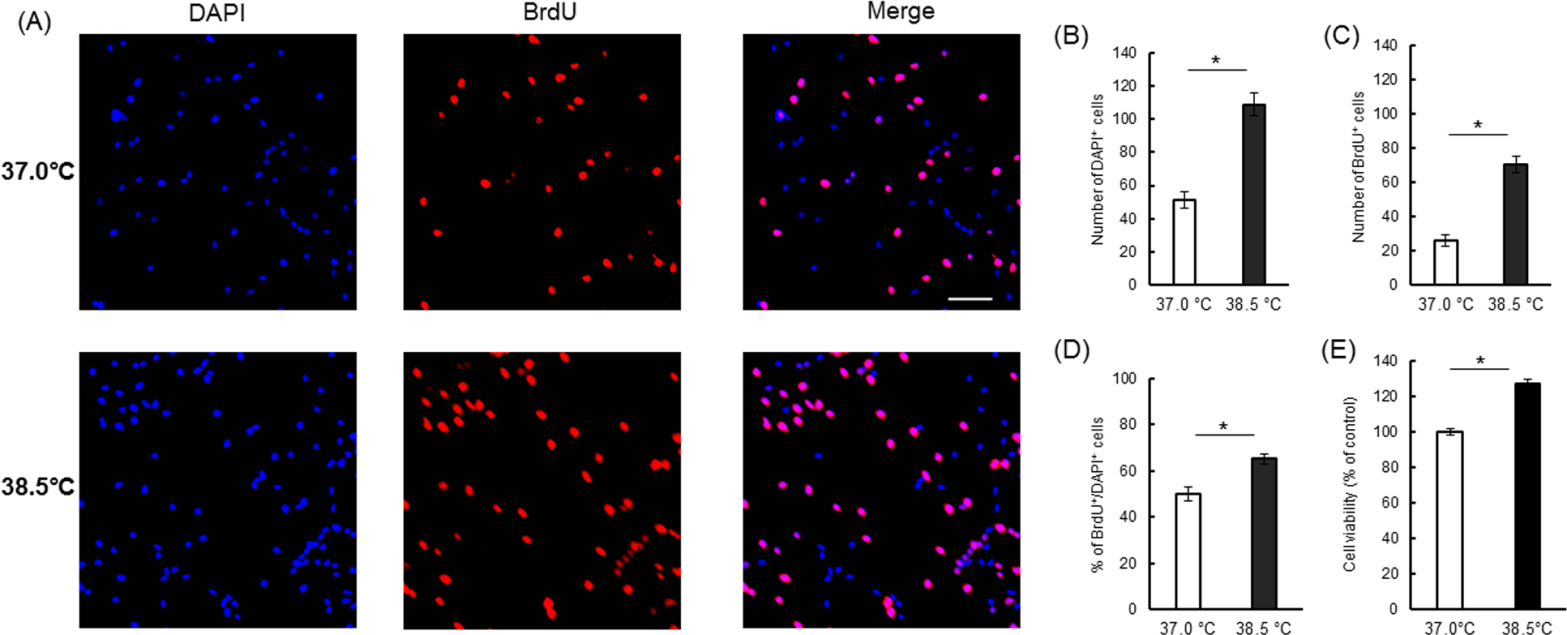
Proliferative effects of mild heat exposure on NSCs/NPCs in a monolayer culture. (A) Representative images of BrdU-labelled cells (red) in control (37.0°C) and heat-exposed (38.5°C) conditions. Nuclei are counterstained by DAPI (blue). Scale bar (white) is 50 μm. Bar diagrams showing the number of total cells (B), number of BrdU^+^ cells (C) and percentage of BrdU^+^/DAPI^+^ cells (D) at 37.0°C and 38.5°C on day 3 of monolayer attached NSC/NPC culture in PM. Results are expressed as mean ± SEM of three independent experiments. *P < 0.05 vs. control condition. (E) On day 4 of monolayer culture in PM, cell viability was measured by MTS assay. Data are expressed as percentages of the control condition (37.0°C). Results are mean ± SEM of three independent experiments. *P < 0.05 vs. control condition.

To evaluate the effects heat exposure on NSC/NPC proliferation, we compared cell viability, neurosphere size, and cell count between cultures grown at control temperature (37.0°C) and 38.5°C under the proliferation condition (PC) (Fig 1A and S1 Fig). Four days of continuous heat exposure (38.5°C) increased viable cell number by about 1.8-fold compared to the control temperature (Fig 1B). Even, in 4 days of culture, initial 1 or 2 days heat exposure followed by culture at 37.0°C (PC1 and PC3) significantly increased viable cell number compared to last 1 or 2 days heat exposure (PC2 and PC4), respectively (S1 Fig). Four days of continuous heat exposure yielded a significant increase in the number of large neurospheres (100–200 μm) and a decrease in the number of medium neurospheres (50–100 μm) (Fig 1D and 1E). The number of cells was also significantly higher after 4 days of continuous heat exposure compared to the control temperature (Fig 1C). Also, cell viability (Fig 2E) and total number of cells (DAPI^+^ cells; Fig 2A and 2B) in monolayer attached cultures were significantly increased by mild heat exposure (38.5°C). In addition, the number of BrdU^+^ cells (Fig 2A and 2C) and percentage of BrdU^+^ cells to total cells (Fig 2A and 2D) were significantly higher at 38.5°C.

### Effects of mild heat exposure on HSPs induction

**Fig 3:**
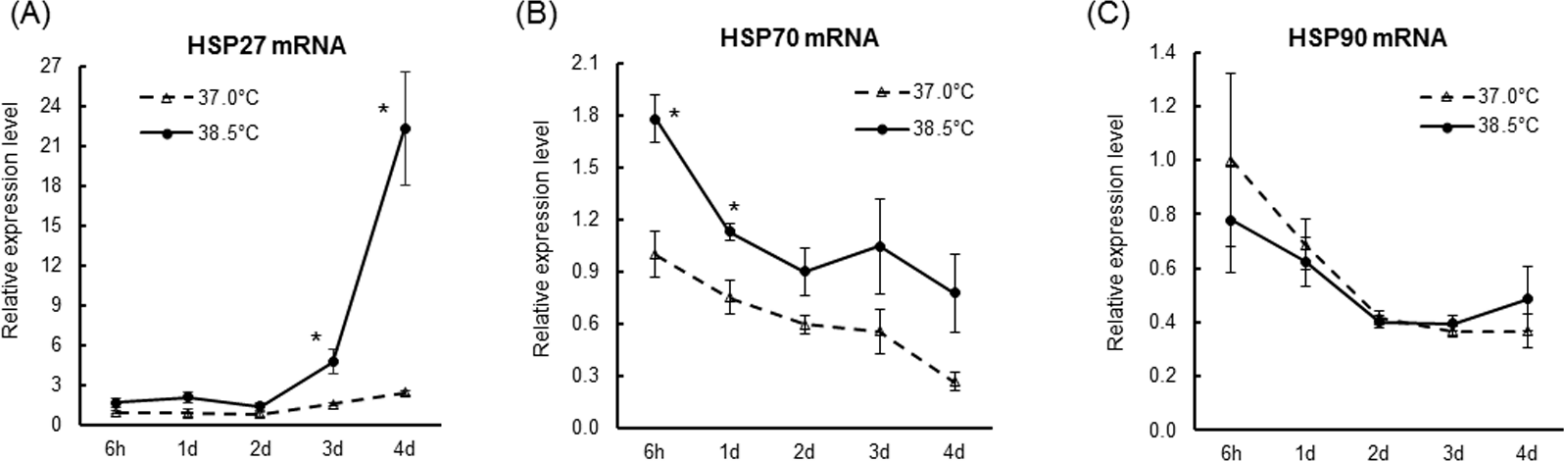
Mild heat exposure upregulates HSP27 mRNA expression. NSCs/NPCs were cultured at 37.0°C or 38.5°C for the times indicated. Total RNA was prepared from each culture and cDNA was synthesized and subjected to real-time PCR using specific primers for HSP27 (A), HSP70 (B) and HSP90 (C). 18S rRNA was used as an internal control. Values are mean (±SEM) fold-increase in the ratio of each gene to 18S rRNA, with the value at 37.0°C/6 h set to 1.0. *P < 0.05 versus control temperature.

To identify the mechanisms underlying increased NSC/NPC proliferation under heat exposure, we first examined effects on expression of HSPs, critical prosurvival (anti-apoptotic) proteins under cellular stress [31–35]. Real-time PCR showed that HSP70 mRNA level decreased with time during NSC/NPC culture at the control temperature (Fig 3B), whereas HSP27 mRNA expression did not change (Fig 3A). Heat exposure increased HSP70 mRNA level during the earlier phase (Fig 3B) while HSP27 mRNA level did not change during this period. Starting from day 3 and continuing to day 4 of culture; however, HSP27 mRNA level was increased to 9-fold in cells grown at 38.5°C than at 37.0°C (Fig 3A). On the other hand, heat exposure had no significant effect on HSP90 mRNA expression (Fig 3C).

### Mild heat exposure elevated BDNF mRNA and CREB phosphorylation

**Fig 4:**
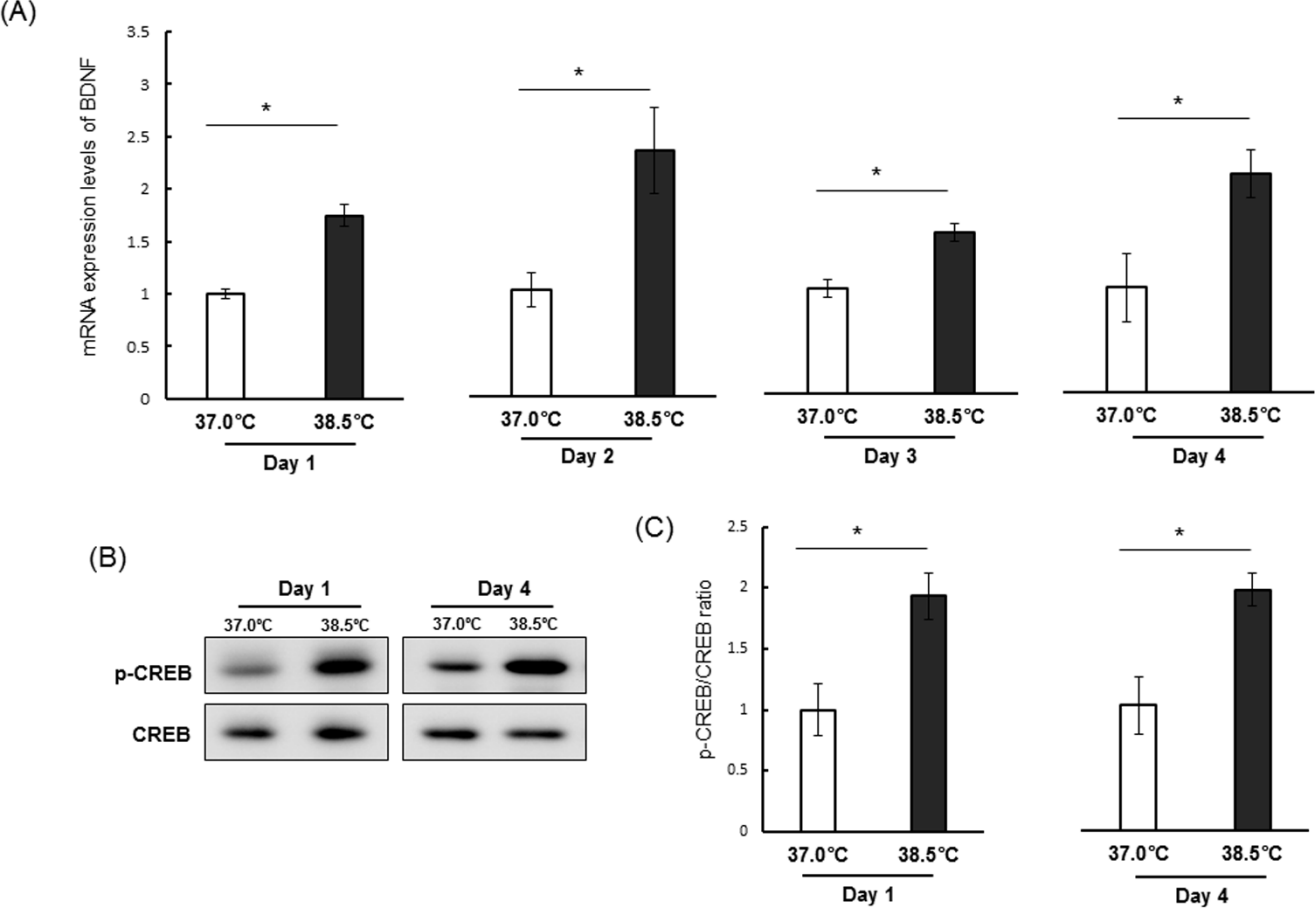
Upregulation of BDNF transcription and CREB phosphorylation by mild heat exposure. (A) Relative mRNA expression levels of BDNF on day 1 to day 4 at 37.0°C and 38.5°C. Values are mean (±SEM) fold-increase in BDNF to 18S rRNA ratio. 18S rRNA was used as an internal control. Values at 37.0°C were set to 1.0. *P < 0.05 versus control temperature. Representative blots (B) and mean relative blot density (C) showing expression of phospho-CREB and CREB in proliferating NSCs/NPCs on day 1 and day 4 of mild heat exposure. The blot intensity of phospho-CREB in each group was normalized to that of CREB and the values at 37.0°C were set to 1.0. Values are mean ± SEM of three independent experiments. *P < 0.05 vs. control temperature.

To further explore the mechanisms for enhanced proliferation, we examined whether heat exposure upregulates expression of BDNF, a neurotrophic factor. Real-time PCR demonstrated that mild heat exposure significantly increased BDNF mRNA level in NSCs/NPCs at all time points from day 1 to day 4 compared to the control temperature (Fig 4A). It has been shown that BDNF expression is directly dependent on the phosphorylation of cAMP response element binding protein (CREB) [43]. Thus, we assessed whether heat exposure also alters the level of p-CREB. Similar to BDNF mRNA, heat exposure significantly increased p-CREB levels in NSCs/NPCs both on day 1 and day 4 (Fig 4B and 4C).

### Mild heat exposure increased intracellular ROS level

**Fig 5:**
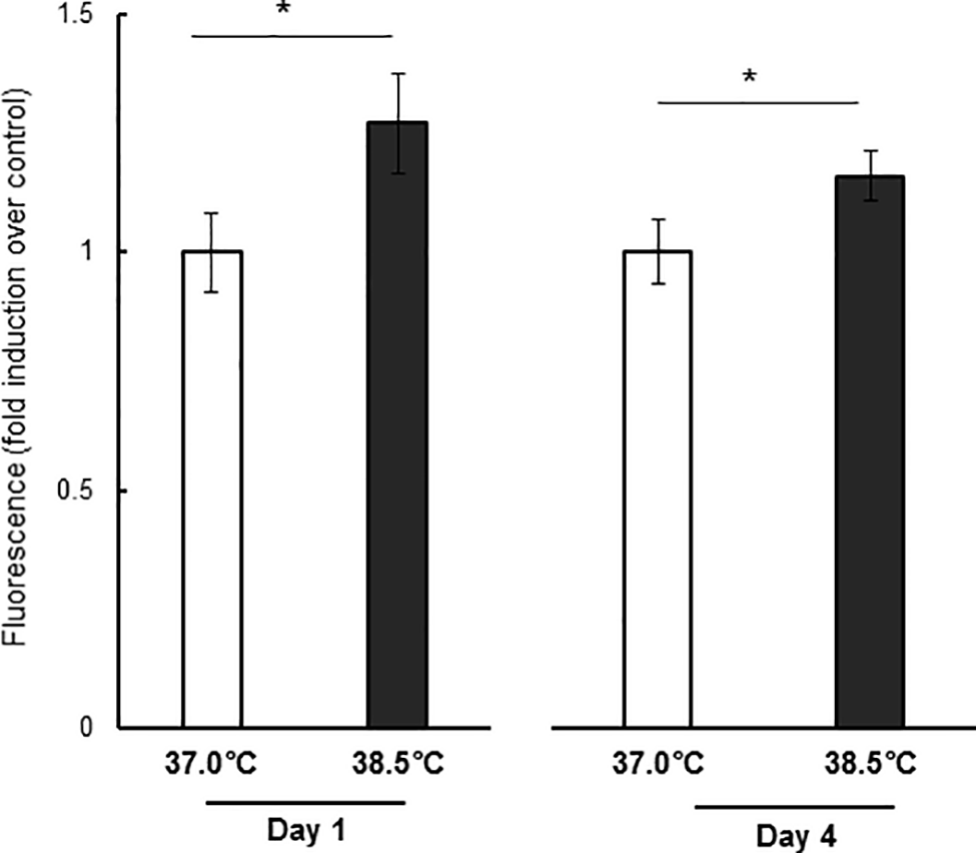
Effects of mild heat exposure on intracellular ROS level. Values are mean ± SEM of four independent experiments showing relative intracellular ROS levels in control (37.0°C) or heat-exposed (38.5°C) NSCs/NPCs on day 1 and day 4. The values at 37.0°C were set to 1.0. *P < 0.05 vs. control temperature.

Hyperthermia has been reported to induce ROS production in various cell types [44–47]. In contrast to the damaging effects of ROS, there is evidence that in some systems, especially in NSCs/NPCs, nontoxic ROS levels can actually promote cell proliferation and survival [48–50]. Thus, we measured intracellular ROS levels in NSCs/NPCs at the control culture temperature and under mild heat exposure. In heat-exposed cells, intracellular ROS levels were increased nearly 27% on day 1 compare to control cells and were still approximate 16% higher on day 4 (Fig 5).

### Mild heat exposure promoted Akt activation

**Fig 6:**
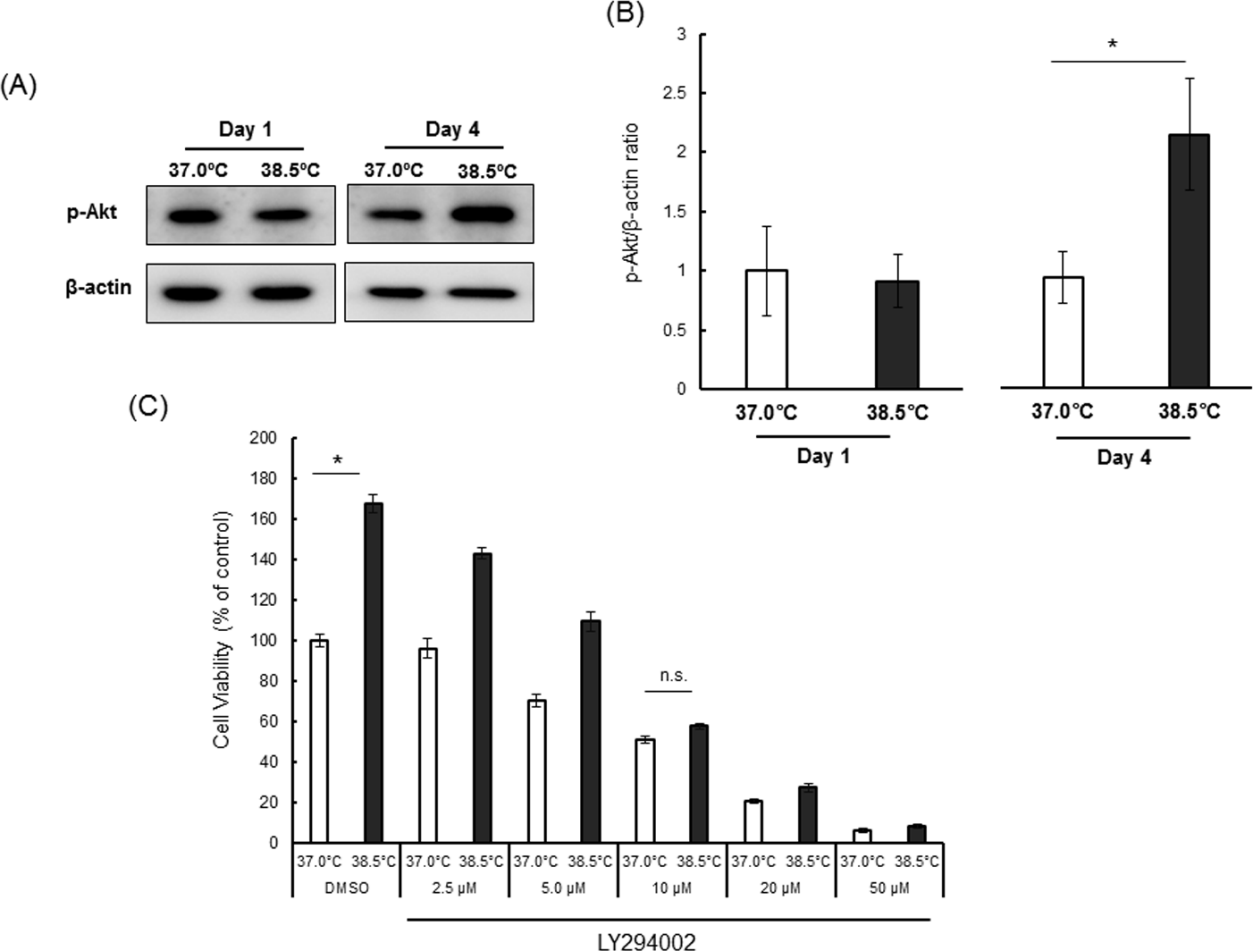
Phospho-Akt (p-Akt) upregulation in neural stem/progenitor cells (NSCs/NPCs) by mild heat exposure. Representative blots (A) and mean relative blot density (B) showing expression of phospho-Akt in NSCs/NPCs on day 1 and day 4. The blot intensity of phospho-Akt in each group was normalized to that of β-actin and the values at 37.0°C were set to 1.0. Values are mean ± SEM of three independent experiments. *P < 0.05 vs. control temperature. 6 (C) Viability of NSCs/NPCs at 37.0°C and 38.5°C in the presence of the PI3K inhibitor LY294002 (0−50 μM) or vehicle (DMSO). Viability of cells in DMSO at 37.0°C was taken as control, and data are expressed as percentages of the control condition. Results are mean ± SEM of three independent experiments. *P < 0.05 vs. control condition.

Mild heat stress has been reported to induce phosphorylation and activation of Akt/PKB in NIH3T3 fibroblasts [51]. Moreover, BDNF [52, 53] and ROS [53], both induced by mild heat exposure, have been reported to activate Akt in NSCs/NPCs. In addition, HSP27 has been suggested to be important for sustained Akt activity [36–38]. Thus, we examined whether mild heat exposure changes Akt phosphorylation (activation status) in NSCs/NPCs. The level of phosphorylated Akt did not change upon heat exposure on day 1, but continuous mild heat exposure significantly increased p-Akt levels on day 4 (Fig 6A and 6B).

#### Heat exposure promoted NSC/NPC proliferation in a PI3K/Akt-dependent manner

Treatment with the PI3K inhibitor LY294002 (LY) decreased Akt phosphorylation (S2 Fig) and abolished the enhanced cell viability induced by heat exposure, as measured by MTS assay (Fig 6C). Treatment with LY dose-dependently inhibited cell viability in both control and heat-exposed conditions. At 2.5 μM, LY treatment decreased cell viability by approximately 4% at 37.0°C and 14% at 38.5°C, whereas 5.0 μM LY reduced cell viability by 30% at 37.0°C and 35% at 38.5°C. There was no significant difference in cell viability between the groups (37.0°C and 38.5°C) at 10, 20, or 50 μM LY. Similar results were also observed in neurosphere growth (S2 Fig). These results suggested that enhancement of NSC/NPC proliferation by heat exposure may be mediated through the PI3K/Akt pathway.

### Mild heat exposure promoted neural differentiation

**Fig 7:**
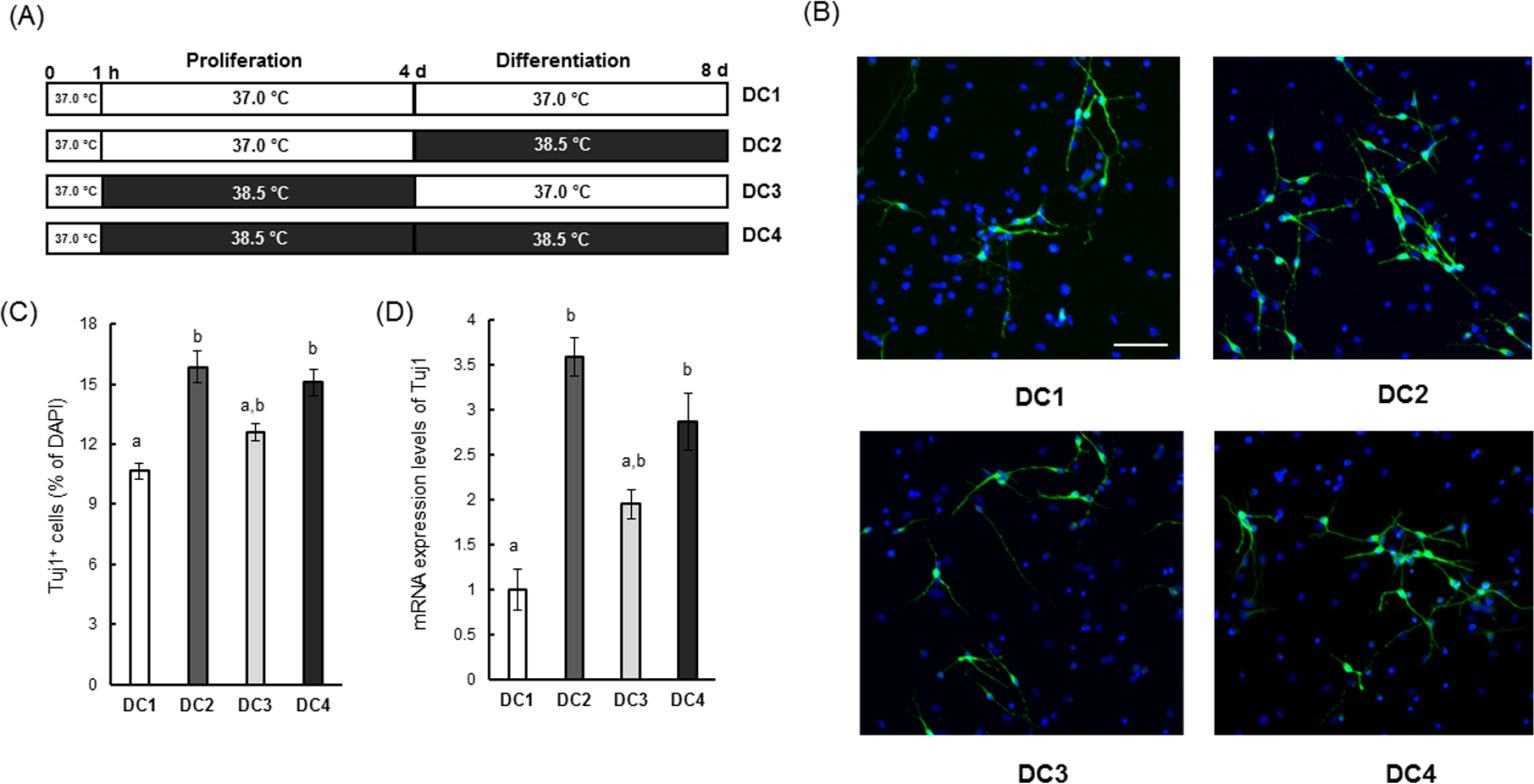
Neuronal differentiation of NSCs/NPCs induced by mild heat exposure. (A) Culture of NSCs/NPCs under four different conditions. (B) Representative images of Tuj1-positive cells (green) on day 4 of differentiation. Nuclei were counterstained with DAPI (blue). White bar indicates 50 μm. (C) Quantification of Tuj1-positive cells. Data are expressed as percentage of total cells. Values are mean ± SEM of four independent experiments. Bars not sharing a common letter are significantly different at P < 0.05 by one-way ANOVA, followed by Tukey’s HSD for post-hoc comparisons. (D) Relative mRNA expression levels of Tuj1 on day 4 of differentiation. Values are the mean (±SEM) fold-increase in Tuj1 to 18S rRNA (internal control) ratio. The values of control temperature group were set to 1.0. Bars not sharing a common letter are significantly different at P < 0.05 by one-way ANOVA followed by Tukey’s HSD for post-hoc comparisons.

In our previous study, we reported that most of the heat-induced newborn cells in the hypothalamus of LTHA rats were co-labeled with a neuronal marker rather than glial markers, indicating that heat exposure mainly promotes neuronal differentiation [21]. To examine whether this effect is mediated directly by heat, we compared neuronal marker expression between control and heat-exposed cultures under the four conditions specified in Fig 7A; control temperature during both proliferation and differentiation (Differentiation Condition 1; DC1), control temperature during proliferation and mild heat exposure during differentiation (DC2), mild heat exposure during proliferation and control temperature during differentiation (DC3), and mild heat exposure during both proliferation and differentiation (DC4). On day 4 in the differentiation condition, nearly 10% of cells maintained in the DC1 were identified as neuron by staining the cells with anti-Tuj1 (a mature neuron marker) antibody (Fig 7B and 7C). Mild heat exposure under differentiation conditions (DC2 and DC4) increased the percentage of Tuj1-positive cells by approximately 5%, indicating that mild heat directly promotes neuronal differentiation of NSCs/NPCs (Fig 7B and 7C). In addition, we also investigated the effects of previous proliferation condition temperature (control or mild heat exposure) on NSC/NPC differentiation. The percentage of Tuj1-positive cells in cultures subjected to mild heat exposure under the proliferation condition and control temperature under the differentiation condition (DC3) did not differ significantly compared to DC1. However, the percentage of Tuj1-positive cells in DC4 was significantly higher compared to DC1 (Fig 7B and 7C). These results indicate that heat exposure during proliferation only is not sufficient to promoting neuronal differentiation. This finding was consistent with expression levels of Tuj1 mRNA during these various conditions (Fig 7D).

## Discussion

In this study, we demonstrated that larger neurospheres, a greater number of total cells and increased cell viability in a cortical NSC/NPC culture at 38.5°C compared to control, 37.0°C (Fig 1). Moreover, the number of total cells and BrdU^+^ cells in monolayer attached NSCs/NPCs culture were significantly increased in the heat-exposed condition (38.5°C; Fig 2). These data strongly suggested that mildly elevated culture temperatures can increase the rate of NSC/NPC proliferation. Tissue temperatures increase by a few degrees Celsius during heat acclimation or under febrile conditions. Exposure of rats to a constant high ambient temperature (T_a_) of ~32.0°C elevated hypothalamic temperature (T_hy_) compared to control rats maintained at a T_a_ of ~24.0°C [54]. In previous studies, we also demonstrated that NSC/NPC proliferation in the hypothalamic area was significantly increased in rats exposed to 32.0°C (heat exposed; HE) ambient temperature compared with control rats maintained at a T_a_ of 24.0°C [21–23]. Accordingly, we speculated that proliferation of NSCs/NPCs *in vitro* may be enhanced by a mild increase in temperature. In this current study, we showed that a +1.5°C increase in culture temperature significantly increased the proliferation of cortical NSCs/NPCs isolated from rat fetuses. However, NSCs/NPCs from different brain regions may behave differently in culture. It will be interesting to see if NSCs/NPCs from other parts of the brain like hypothalamus respond similarly to heat exposure.

The effects of heat exposure on cell growth depend on multiple factors including cell type, culture condition, magnitude of temperature elevation (from normal), and exposure time [55]. High temperature has been shown to act as a proteotoxic stress, and exerts a variety of anti-proliferative effects on mammalian cells including p21-dependent cell cycle arrest [56–58] and MAPK-dependent apoptosis [59]. Proliferation of cultured NSCs/NPCs was also inhibited by severe heat stress (42–44°C) [60]. While severe heat shock leads to apoptosis, mild heat exposure has been reported to stimulate the growth of various cell types [25–27]. In this study, 38.5°C (mild heat exposure) accelerated proliferation and induced HSP27 expression (Fig 3A). HSP27 has been reported to directly block release of pro-apoptotic factors from mitochondria [61, 62]. HSP27 can also interact with Akt, and this interaction has been suggested to sustain Akt activity [36–38]. In this study, transcriptional induction of HSP27 began to increase from day 3 (Fig 3A) and rose dramatically on day 4, concomitant with increased p-Akt (Fig 6A and 6B). Hence, HSP27 may act as a key modulator of heat-induced NSC/NPC proliferation through Akt activation.

However, mild heat does not appear to induce cell proliferation under non-growth conditions but rather facilitates growth factor-stimulated cell proliferation [25]. PI3K/Akt signaling has been implicated in growth factor-induced NSC/NPC proliferation [39, 63]. In this study, the proliferative effects of mild heat exposure were suppressed by a PI3K inhibitor, suggested involvement of the PI3K/Akt pathway (Fig 6C, S2 Fig). In a previous study, ROS^hi^ NSCs/NPCs were shown to be hyperproliferative through a ROS- and PI3K/Akt-dependent pathway [53]. Similarly, heat-exposed NSCs/NPCs exhibited slightly elevated intracellular ROS level (Fig 5), Akt activation (Fig 6), and NSC/NPC proliferation. Although we did not investigate about how Akt activation increases NSC/NPC proliferation, other studies suggest that modulation of Wnt signaling through GSK3β could be involved in this process [64].

Several transcription factors, enzymes, and cytokines are implicated in the regulation of NSC/NPC proliferation and differentiation. For example, CREB has been shown to be important for the survival and expansion of mouse neural progenitor cells [65]. In this study, we found that mild heat exposure elevated CREB phosphorylation and BDNF mRNA expression (Fig 4), in accordance with previous studies showing elevated BDNF in heat acclimated rats [23] and mice [66]. BDNF transcription is dependent on CREB phosphorylation [43]. In the presence of other growth factors, BDNF stimulates NSC/NPC proliferation in a ROS-dependent manner through Akt activation [53]. BDNF has also been reported to play a critical role in cultured motoneuron survival through activation of the PI3K-Akt pathway [67]. Although it is not clear how BDNF and p-CREB are induced by heat, BDNF may play an important role in heat-induced NSC/NPC proliferation. Activation of CREB and increased expression of BDNF mRNA were observed after only one day of heat exposure in growth media (Fig 4), substantially sooner than the other responses such as HSP27 and p-Akt upregulation. Thus, these responses may be upstream signaling events in an Akt-dependent proliferation pathway. It will be interesting to investigate that how heat exposure modulates CREB activation and BDNF expression.

Our previous studies revealed that heat exposure promotes mainly neural differentiation [21, 22, 24]. In this study, we also found that heat exposure in the absence of growth factors enhanced the proportion of cells positive for Tuj1, a marker of mature neurons (Fig 7). In our previous study, constant heat exposure was required for promoting neuronal differentiation of heat-induced newborn cells in the rat hypothalamus [21], and the current study indicated that heat exposure during differentiation (but not proliferation) is essential for promoting neural differentiation of NSCs/NPCs (Fig 7). It remains to be determined if heat exposure promotes differentiation of NSCs/NPCs into other cell types (astrocytes and oligodendrocytes). A detailed study focusing on differentiation of NSCs/NPCs into various cell types and mechanism of differentiation under heat exposed condition is required.

**Fig 8:**
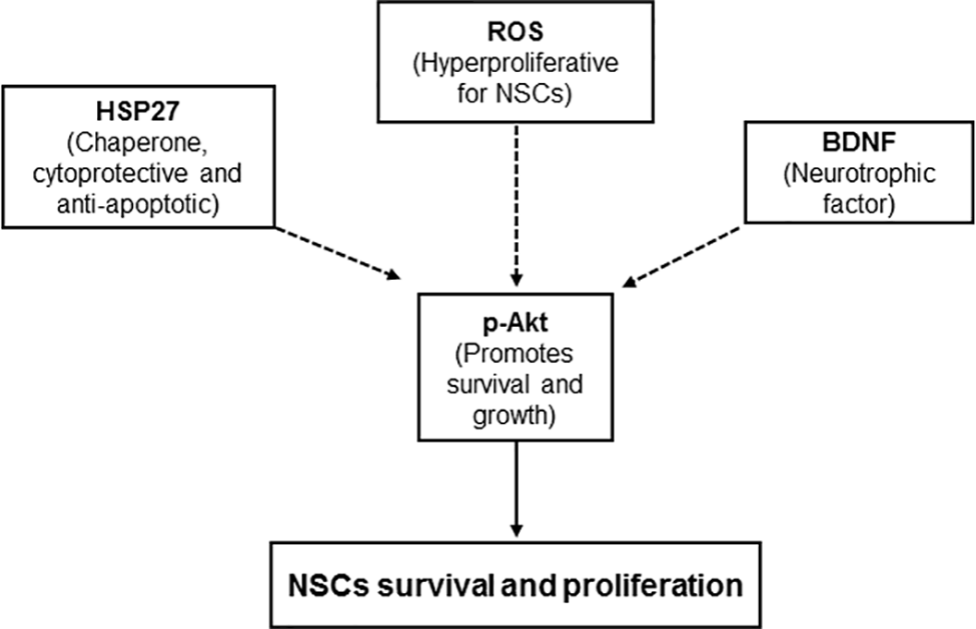
Schematic diagram of possible mechanisms for heat-induced proliferation of neural stem/progenitor cells.

In conclusion, treatment with mild heat enhanced the proliferation and neural differentiation of NSCs/NPCs *in vitro*. This study provides a hint that the direct effect of temperature may underlie the heat-induced neurogenesis in rat hypothalamus associated with heat acclimation. This enhanced NSC/NPC proliferation by mild heat exposure may be mediated by Akt activation through upregulation of BDNF and HSP27 as well as by slight elevation of intracellular ROS (Fig 8).

## Supporting information

S1 Fig Cell viability, cell count, and neurosphere size distribution under different conditions of heat exposure. (A) NSCs/NPCs were cultured in proliferation media (PM) under different heat exposure conditions. Proliferation at 37.0°C was used as the control. (B) On day 4 of culture in PM, viability of NSCs/NPCs was measured by MTS assay in each condition. Data are expressed as percentages of the control. Values are mean ± SEM of five independent experiments. *P < 0.05. (C) On day 4, the neurospheres formed under different conditions were dissociated separately by mild agitation and the total number of cells was counted by trypan blue exclusion. Data represent mean ± SEM of five independent experiments. (D) Representative images of neurospheres formed under different heat exposure conditions on day 4 of proliferation. Bar indicates 250 μm. (E) Quantitative analysis of neurosphere distribution arbitrarily divided into four classes according to diameter. Data are percentages of the total number of neurospheres. Values are mean ± SEM of four independent experiments.
(TIF)

S2 Fig Effects of heat exposure on neurosphere growth and Akt activation in presence of LY294002. (A) The calculated area (μm^2^) of neurospheres per well at 37.0°C and 38.5°C in the presence of different concentrations of the PI3K inhibitor LY294002. Data are expressed as percentages of the control condition (37.0°C in DMSO). Results are mean ± SEM of three independent experiments. *P < 0.05 vs. control condition. Representative blots (B) and mean relative blot density (C) showing expression of phospho (p)-Akt in NSCs/NPCs treated with LY294002 (10 μM) or DMSO. The blot intensity of phospho-Akt in each group was normalized to that of β-actin, and the values at 37.0°C in DMSO were set to 1.0 as the control condition. Values are mean ± SEM of three independent experiments. *P < 0.05 vs. control condition.
(TIF)

S1 Table List of primers for qPCR. (DOCX)
