# Supplementary figures and images for: Direct exposure to mild heat promotes proliferation and neuronal differentiation of neural stem/progenitor cells in vitro
Source: PLoS One. 2017 Dec 29;12(12):e0190356. doi: 10.1371/journal.pone.0190356 (PMC5747471; doi:10.1371/journal.pone.0190356)

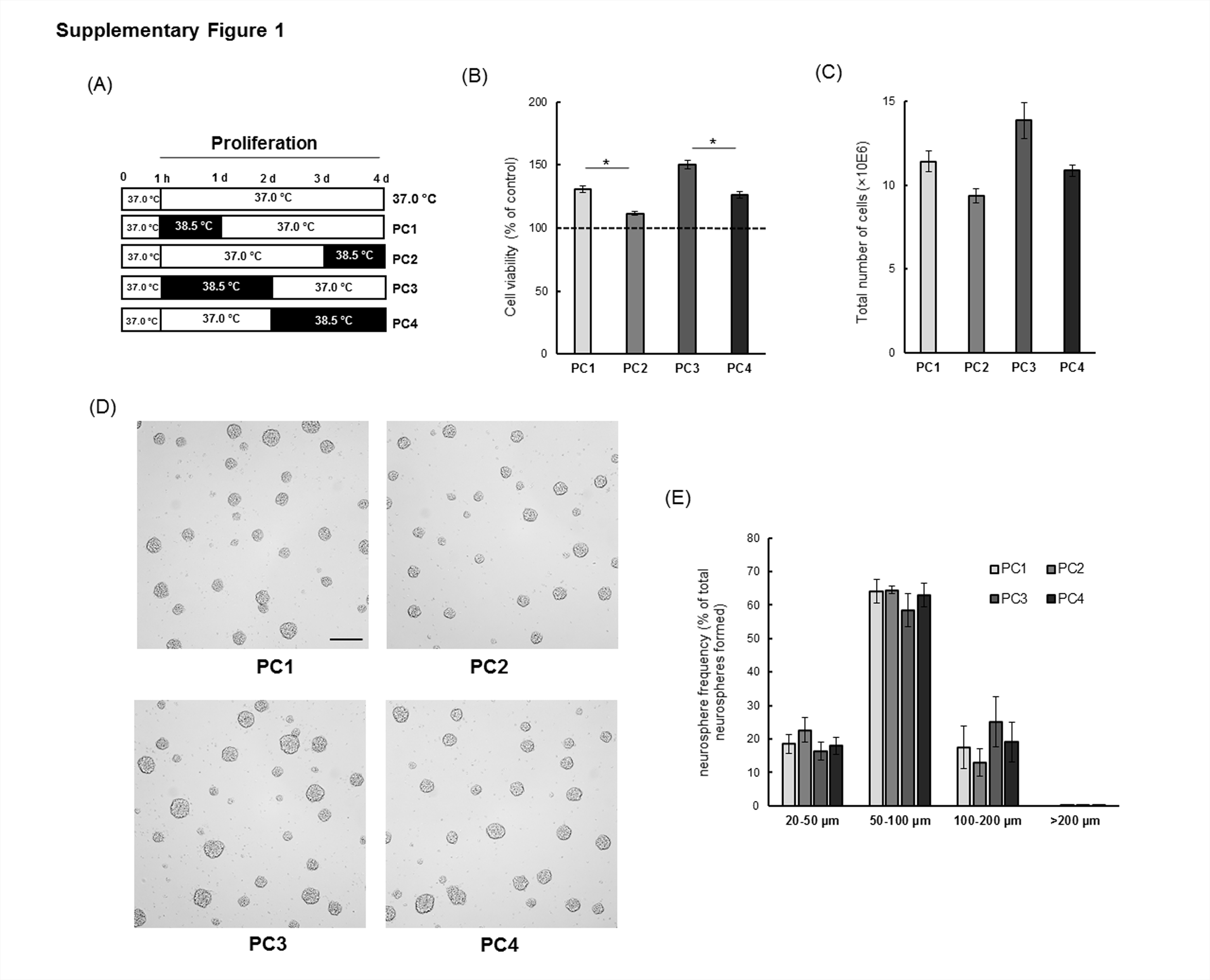

Supplement: S1 Fig — (A) NSCs/NPCs were cultured in proliferation media (PM) under different heat exposure conditions. Proliferation at 37.0°C was used as the control. (B) On day 4 of culture in PM, viability of NSCs/NPCs was measured by MTS assay in each condition. Data are expressed as percentages of the control. Values are mean ± SEM of five independent experiments. *P < 0.05. (C) On day 4, the neurospheres formed under different conditions were dissociated separately by mild agitation and the total number of cells was counted by trypan blue exclusion. Data represent mean ± SEM of five independent experiments. (D) Representative images of neurospheres formed under different heat exposure conditions on day 4 of proliferation. Bar indicates 250 μm. (E) Quantitative analysis of neurosphere distribution arbitrarily divided into four classes according to diameter. Data are percentages of the total number of neurospheres. Values are mean ± SEM of four independent experiments. (TIF) [file pone.0190356.s001.TIF]

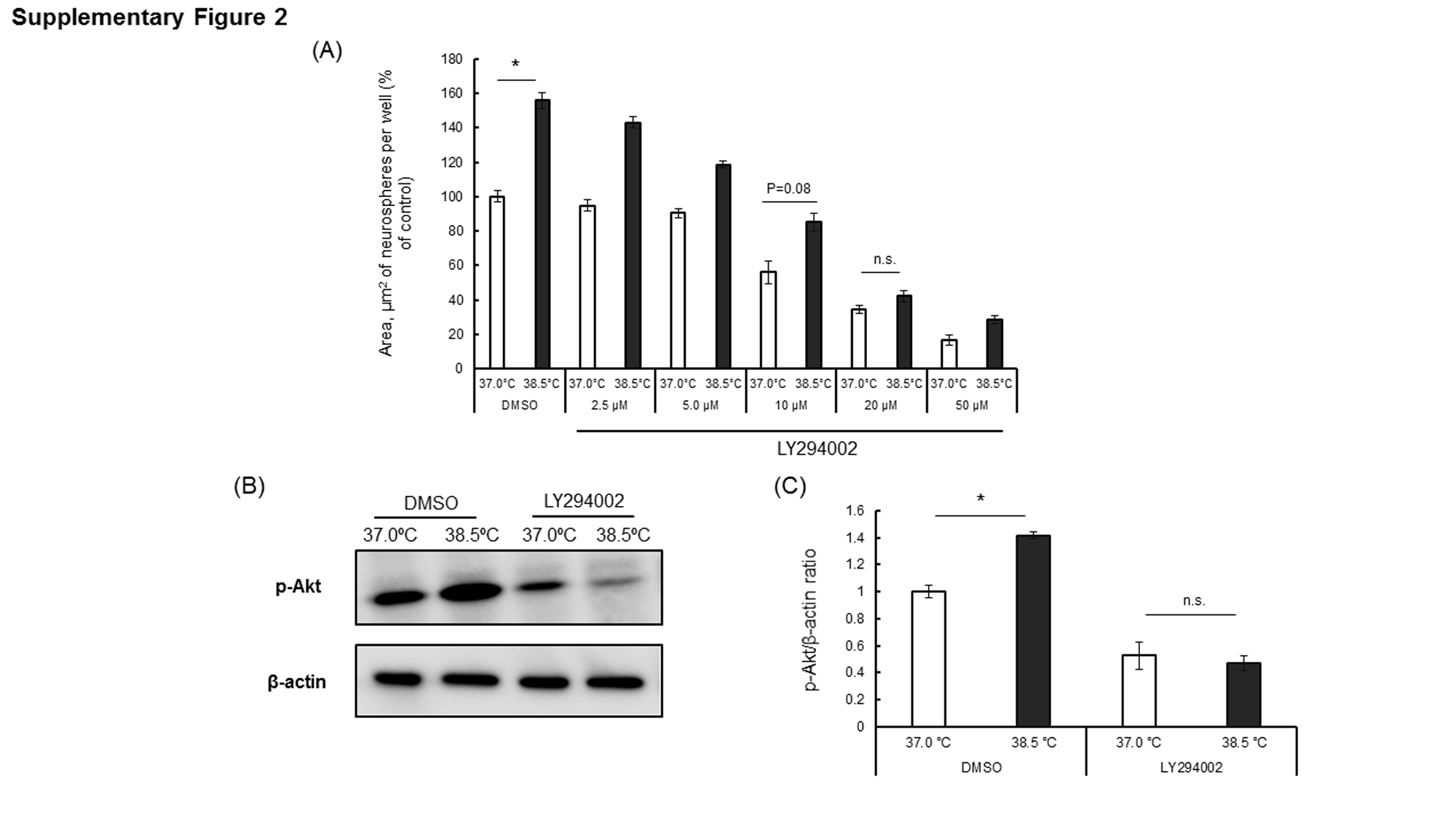

Supplement: S2 Fig — (A) The calculated area (μm2) of neurospheres per well at 37.0°C and 38.5°C in the presence of different concentrations of the PI3K inhibitor LY294002. Data are expressed as percentages of the control condition (37.0°C in DMSO). Results are mean ± SEM of three independent experiments. *P < 0.05 vs. control condition. Representative blots (B) and mean relative blot density (C) showing expression of phospho (p)-Akt in NSCs/NPCs treated with LY294002 (10 μM) or DMSO. The blot intensity of phospho-Akt in each group was normalized to that of β-actin, and the values at 37.0°C in DMSO were set to 1.0 as the control condition. Values are mean ± SEM of three independent experiments. *P < 0.05 vs. control condition. (TIF) [file pone.0190356.s002.TIF]
