# Supplementary material for: Direct exposure to mild heat promotes proliferation and neuronal differentiation of neural stem/progenitor cells in vitro
Source: PLoS One. 2017 Dec 29;12(12):e0190356. doi: 10.1371/journal.pone.0190356 (PMC5747471; doi:10.1371/journal.pone.0190356)
Supplement: S1 Table — (DOCX) [file pone.0190356.s003.docx]

| Genes | Forward Sequence | Reverse Sequence |
| --- | --- | --- |
| BDNF | AGCTGAGCGTGTGTGACAGT | ACCCATGGGATTACACTTGG |
| HSP27 | CTGGACGTCAACCACTTCG | AGCACCGAGAGATGTAGCC |
| HSP70 | AACGTGCTGCGGATCATCAAC | GCTTGTTCTGGCTGATGTCCT |
| HSP90 | TGAGGCAGAGGAAGAGAAAGG | AGTGCTTGACTGCCAAGTGGT |
| Tuj1 | GGCCTTTGGACACCTATTCA | TGCAGGCAGTCACAATTCTC |
| 18S rRNA | GCAATTATTCCCCATGAACG | GGCCTCACTAAACCATCCAA |

S1 Table: List of primers for qPCR
